# Importance of Breast Sensation After Mastectomy: Evidence from Three Sources

**DOI:** 10.1089/whr.2023.0106

**Published:** 2023-12-04

**Authors:** Stacy T. Lindau, El A. Pinkerton, Emily M. Abramsohn, Charles M. Fuller, Danielle Grubb, Tania Mendoza, Amy K. Siston

**Affiliations:** ^1^ Department of Obstetrics and Gynecology, The University of Chicago, Chicago, Illinois, USA.; ^2^ Department of Medicine-Geriatrics, The University of Chicago, Chicago, Illinois, USA.; ^3^ Department of Psychiatry and Behavioral Neuroscience, The University of Chicago, Chicago, Illinois, USA.

**Keywords:** breast sensation, breast sensory function, sexual function, breast cancer, breast reconstruction, mastectomy

## Abstract

**Background:**

Every year, more than 90,000 U.S. women undergo mastectomy. More than 40% have reconstruction. Following reconstruction, most women experience persistent partial or complete numbness of the reconstructed breasts, and many experience pain. Yet, breast reconstruction procedures focus largely on esthetic outcomes with mixed impact on sensory outcomes and little attention to pain. This study examines whether and how breast sensation is important to women.

**Materials and Methods:**

Conventional content analysis of extant qualitative data from a clinical registry (29 women with prior breast surgery for cancer, 2008–2022), a volunteer community sample (qualitative interviews with 6 women with and 5 without breast cancer, 2019), and from a Twitter social media survey (*N* = 32, 2022).

**Results:**

Functions of the breast identified by women with and without cancer include breastfeeding, sexual function, and femininity. Five interrelated themes on the importance of breast sensation emerged among women with breast cancer history: sexual function, experience of partnered sex or relationship with one's sexual partner, breast embodiment, effect of breast pain on sexual function, and importance to psychological wellbeing. Women, advocates, and clinicians described a lack of patient–physician communication in this domain that exacerbates the negative impact of breast sensation loss on health and wellbeing.

**Conclusions:**

Breast sensation is important to women following mastectomy, yet a gap exists in patient–physician communication about the impact of mastectomy and reconstruction on breast sensory function. Lessons for physicians, scientists, and skeptics are conveyed about why the basic integrity of women's bodies matters for practice and science.

## Introduction

In the United States alone, more than 90,000 women have one or both breasts removed for breast cancer treatment or risk reduction each year.^1,2^ Of these, more than 40% undergo breast reconstruction procedures^3^ and up to 60% experience persistent breast numbness.^4–7^ Loss of breast sensation following mastectomy has been associated with decreased sexual function, decreased quality of life, and increased risk of thermal injury.^8–12^ Twenty-five to 60% of women also experience chronic breast pain following mastectomy, typically from damage to severed intercostal nerves.^13,14^ Chronic breast pain has also been associated with decreased quality of life, depression, sleep disturbance, and decreased general health following mastectomy.^15,16^

Innovations in breast reconstruction surgery and related patient counseling have focused largely on esthetic outcomes. Surgical interventions to preserve sensory function—including skin- and nipple-sparing procedures, nerve coaptation, and autologous flaps—are evolving rapidly.^6,17–22^ While many show promise, they are available only to a subset of patients, pain is not typically a target, and sensory outcomes have been mixed and often disappointing.^6,17,18,23,24^ Even women who are satisfied with the esthetic outcome of reconstruction express distress regarding lack of communication with the surgeon about loss of breast sensation and implications for sexual function and other aspects of health and quality of life.^25,26^ Emerging research that better characterizes patient-centered outcomes related to breast sensation represents a promising shift in focus,^27,28^ but evidence is scant that women with lived experience of breast cancer or mastectomy have been included in efforts to innovate solutions to loss of breast sensation following mastectomy.

In contrast, sexual function is recognized as an important patient-centered outcome of prostate cancer treatment and is routinely addressed in medical decision making and treatment planning.^29^ Research funded by the National Institutes of Health has led to new interventions to promote sexual function outcomes for prostate cancer patients. Breakthroughs in surgical technique for penile cancer (<0.001% prevalence rate) and genital trauma have enabled five penis transplants worldwide (two of which have required explantation as of this writing).^30^ Johns Hopkins Medicine, where a successful penis and scrotum transplant was performed in 2018, identifies several functional goals of transplant: “urination, restoration of sexual function, and restoration of the patient's sense of wholeness and self.”^31^

The Bionic Breast Project joins patients; clinicians; social and biological scientists; and electrical, mechanical, and molecular engineers to preserve and restore breast function and alleviate pain using bionic technologies among mastectomy patients. This paradigm-shifting approach involves adaptation of technology that imbues a prosthetic hand with a sense of touch and has also been shown to mitigate pain.^32,33^ The Bionic Breast Project has been developed with extramural research support, including funding from the National Cancer Institute. Despite enthusiasm about the concept from patients and clinicians, and growing recognition of the problem of breast sensation loss in the literature, a recent proposal for federal research funding from our group received the following comment from a peer reviewer who questioned the scientific significance of the project by noting as a weakness that “it's not clear how important sensation is to patients after mastectomy.”

This critique touched a nerve (no pun intended) and, combined with ongoing evidence of harm resulting from breast numbness after mastectomy, was a call to bring forth new sources of evidence about the importance of breast sensation. A study was conducted to further establish whether and how breast sensation is important to mastectomy patients and others with concern for this population.

## Materials and Methods

Data from three extant sources were analyzed.

### Registry: Patients with a breast cancer history and sexual function concerns

All patients seen for care at the University of Chicago Program in Integrative Sexual Medicine for Women and Girls with Cancer (PRISM) since October 2008 were eligible to join a prospective research registry. All participants (*N* = 387 as of June 2022) provided written documentation of the informed consent process. The Registry was approved by the University of Chicago Institutional Review Board (IRB).

A qualitative dataset was produced by abstraction of narrative text from clinical progress notes of 109 registrants with a history of surgical treatment for breast cancer. Research coders had no direct interaction with registrants during the data analysis phase. However, all registrants were past or current patients of S.T.L. and both D.G. and E.A. may have previously interacted with some registrants while working as clinical patient educators. Two coders (C.F., D.G.) did a first-pass read of transcripts to deductively identify narrative statements (“quotes”) relating to the importance of breast sensation, including pain.^34^ Using conventional content analysis, coders then inductively analyzed the data, allowing for emergent categories.^35^ Following a consensus-building exercise, a codebook was developed and the data were independently coded by two researchers (C.F., D.G.), with adjudication by two others (E.A., S.T.L.). Data analysis continued until inductive thematic saturation was achieved (no new categories emerged from the data).^36^ More than one code could be applied to a single quote. Quotes were annotated using the convention R,X (R = registrant ID, X = treatment type).

### Qualitative interviews: Women with and without a breast cancer history

Between June and September 2019, qualitative interviews were conducted with a different group of sexually active adult women (*N* = 16) with (BC+) and without (BC−) a breast cancer history recruited by email, flyer, and social media. The primary aim of this 2019 study was to develop a measure of breast sensorisexual function.^27^ The hour-long interviews queried conceptual areas related to breast function and breast embodiment^37^ (one's feeling that their breasts belong to their body). As described above for the Registry study, these interviews were conducted with approval from the same IRB and all participants provided written documentation of informed consent.

Eleven participants responded to an interview prompt, “Describe all the things a woman's breasts do,” yielding data of relevance to the present inquiry that have not previously been analyzed. Interviews were conducted in an academic medical center in the presence of two female qualitative researchers trained in cognitive testing, a Master of Public Health-trained interviewer (E.A. or E.P.), and a detailed note taker (E.A., E.P., or D.G.). E.A. and D.G. may have previously interacted with some participants while working as clinical patient educators, but other researchers had no prior relationship with participants. Audiorecorded interviews were transcribed verbatim. Therefore, transcripts were not returned to participants for feedback. Transcripts were analyzed using conventional content analysis by two independent researchers (E.P., T.M.) We note that T.M. was also a trained patient educator in the PRISM clinic. Analysis elicited descriptive open codes that were organized into categories capturing the key concepts.^35^ Several rounds of independent coding and discussion yielded consensus and a final codebook.

Data were analyzed to exemplify existing theory—informed by extant literature and 15 years of specialized clinical experience (S.T.L.)—regarding breast function to achieve *a priori* thematic saturation.^36^ Quotes were annotated with the participant's unique ID (PXX) and breast cancer history (BC+/BC−).

### Twitter survey: Twitter users

In March 2022, S.T.L. tweeted the following to more than 1,400 Twitter followers: “Need help with a research question pertaining to the problem of numb breasts after #mastectomy. A skeptic says, ‘it's not clear how important sensation is to patients after mastectomy.’ What do you say?” All public responses were coded categorically using a process informed by the methods described for analysis of the PRISM Registry data. A first round of discussion and coding was conducted by T.M. and A.S. together. The codes were then reviewed by S.T.L. and iterated together with T.M. and A.S. to achieve consensus.

Microsoft Excel 2016 (Microsoft Corporation, Redmond, WA) was used to organize data and document findings for all three analyses.

## Results

### Patient registry sample: Women with a breast cancer history and sexual function concerns

Relevant narrative in the medical record was identified for 29 of 109 eligible Registry patients, yielding 34 total quotes. All patients identified as women and were 29–72 years old. Most were non-Hispanic White (16/26 with race/ethnicity data) or non-Hispanic Black (7/26) and postmenopausal (19/20 with data). Eight patients had mastectomy without and 10 with reconstruction. Ten had undergone lumpectomy, two with reconstruction. Five interrelated categories indicating the importance of breast sensation emerged: importance to sexual function, including libido, arousal, and orgasm; importance to the relationship with the sex partner or the experience of partnered sex; importance for the patient's sense of breast embodiment; importance of the effects of breast pain or discomfort on the patient's sexual function and wellbeing; and importance to the patient's psychological wellbeing.

In response to the physician's (S.T.L.) questions about sexual function concerns during PRISM clinic visits, patients commonly described ways that loss of breast sensation had a negative impact on sexual arousal and orgasm. One patient explained, “I used to get pleasure from the nipple and now it's just annoying. I find it actively unarousing” (R7, lumpectomy). Another patient recalls that her breasts and nipples were an “important erogenous zone” but now, “I have no feeling there anymore … without that stimulation, I can't get where I need to be” (R11, mastectomy+reconstruction). A third patient described: “Here's the big thing. A lot of my arousal, most came through my nipples. I had skin-sparing surgery, but not nipple sparing because some of the cancer came into the nipple. … I can feel him [spouse] touching my breasts, it does nothing for me. I've lost my main area of arousal” (R18, mastectomy+reconstruction).

One woman presenting for care for sexual function concerns indicated that her breast sensation was never particularly important to sexual functioning. She found loss of breast sensation less distressing: “Breasts functioning in that they are there. There is not a lot of sensation. That's mostly ok. Never a critical aspect of sexual function. It's a bonus, but not critical” (R29, mastectomy+reconstruction).

Patients also commonly described ways that loss of breast sensation impacted partnered sexual interactions. For some, the partner's sexual interest in and interaction with their numb breasts was distressing: “Before that [mastectomy], they [touch to the breast and sexual arousal] were definitely connected, but now it is not. …When you don't feel something and you know someone is touching them, it's a turn-off. It's horrible because he likes my breasts. That's definitely had an effect” (R10, mastectomy).

This patient identified both loss of arousal and inhibition of arousal (or “turn-off”) due to a negative affective experience resulting from her partner deriving pleasure from her numb breasts. Several women articulated that their partner was not bothered by, and was still sexually interested in, their breasts despite loss of sensation. One patient said, “But they [my breasts] are really nothing to me. … He [her partner] wants to feel them, he wants to have that feeling that means so much” (R8, mastectomy+reconstruction).

The above excerpts also give evidence to the importance of breast sensation for embodiment. Several women described feeling disconnected from their breasts. For example, one patient said she felt “very apprehensive to touch the breasts, not because I'm afraid I'll hurt myself but because I don't like the way my breast feels to my hand” (R22, mastectomy+reconstruction). Some women described a disconnect between how the breasts looked and their lack of function. One patient said, “I don't look at my breasts. They look pretty good. But they are really nothing to me … they do nothing for me” (R8, mastectomy+reconstruction).

Some women experienced both loss of pleasurable sensation and chronic pain or discomfort in the breast or chest area that interfered with sexual function and day-to-day social interactions. One patient remarked on both the physical and psychological impact of feeling pain: “I don't want it to be touched. It hurts when I hug somebody. Every time I hug someone, I'm reminded of the breast cancer” (R25, lumpectomy). Another patient noted that she “couldn't get past first base because [her] chest wall was so sensitive” (R19, lumpectomy+ reconstruction).

Less commonly elicited, but equally distressing to some patients, was the impact of lost breast sensation on their psychological wellbeing, including grief or loss. A patient recalled that when her partner touched her affected breast, “I started crying. Not because it hurt physically” (R22, mastectomy+reconstruction). One patient with no sensation in either breast simply said, “I miss it” (R16, mastectomy). Others were angry, especially those who felt that this outcome was not addressed before treatment: “Not one doctor ever talked to me about the sexual aspect of loss of my nipples. That's where most of my arousal comes from” (R18, mastectomy+reconstruction).

### Qualitative interviews from a volunteer community sample: Women with and without a breast cancer history

Of 11 participants who responded to “Describe all the things a woman's breasts do,” all identified as women, 6 had a cancer history, 6 identified as white, and 5 as African American/Black. Three interrelated functions of the breast emerged: breastfeeding, sexual behavior and function, and signaling femininity.

Almost all respondents named breastfeeding as one of the breast's primary functions. As one participant phrased it, “Obviously they feed children, because that's what they're made for” (P01, BC+).

The sexual function of the breast was commonly cited. Women thought of the breasts as “part of the foreplay,” (P03, BC−) and “pleasure zones for your partner and for yourself” (P11, BC+). For many, their breasts were a dominant, even essential, source of arousal during partnered sex; one respondent reported that “I do have a lot of sensation in my breasts which allows me to ease into sexual activity” (P03, BC−). Other participants described how deficits in breast function after mastectomy had impacted their sexual function. For example, one respondent noted that breast stimulation “helps you get aroused and with an orgasm, it helps a lot, or at least it did for me [before mastectomy]” (P02, BC+). Another participant, who lost much of her breast sensation after mastectomy, wondered what kind of breast function she should describe: “My breasts? Or functioning breasts?” (P11, BC+).

Some women described their breasts as a symbol of femininity, influencing their self-image and perception by others: “Growing up I didn't like my breasts, but I've come to like them and accept them as the female part of the body. … It is a symbol of power for me, just because it distinguishes me from the males” (P03, BC−). Another woman spoke to embodiment and the importance of breasts for femininity; “[having breasts is] just part of, I'm a woman! You know, it's a part of me!” (P13, BC−).

### Twitter survey

The original post yielded 12 “likes,” 5 “retweets,” and 32 replies over 12 weeks from people who identified as having had surgical treatment for breast cancer (*n* = 15), physicians (*n* = 6), nurses (*n* = 2), psychologists or other PhD-trained professionals (*n* = 3), and others (*n* = 3). Some identified with more than one group. Replies were categorized as comments on the skeptic's question about whether breast sensation was important to women; effects of lost breast sensation on other symptoms, daily activities, or sexual function; patient–physician communication; and physical and psychological relief.

Several people with a history of surgical treatment for breast cancer commented on the nature of the skeptic's question. One person said, “Take any part of the torso and have it dead, and I think most people would find that pretty important.” Three people commented specifically on the lack of attention to restoring breast function after mastectomy: “Reconstruction, is not reconstruction, it is more like remodeling. Not the same …2 breast mounts after BC [breast cancer], not breasts after cancer.” One “pre-vivor,” reflecting the sentiment of others, asked, “Does that skeptic happen to be male?” Several clinicians also objected that someone would question whether loss of breast sensation would matter to women. A nurse replied, “Makes me want to cry. Of course breast sensation is important. At any age. Grrr,” and a well-known psycho-oncologist commented: “Anyone who has spent 5 minutes having honest conversations with women postmastectomy knows that's BS. Not to mention we have plenty of empirical data reflecting this reality. People may learn to live with all kinds of loss, but for many, it's still a profound loss.”

One man responded: “I'm not a doctor but that's [questioning whether sensation matters to women] pretty much the dumbest thing I've ever heard.”

Others spoke of the importance of sensation for daily activities. A person who had mastectomy with lymphadenectomy wrote, “I literally can't hold something under my right arm, in my axilla, for example, newspaper tucked under, as I forget it's there as I can't feel it and drop it.” A physician caring for women with breast cancer said:

“We also need to remember the importance of a hug, feeling your child against your chest, and proprioception. Beyond sexual health considerations, patients undergoing mastectomies are friends, mothers, and humans who still need to live in their bodies and deserve them to be whole.”

Two people described itching as a bothersome symptom related to breast sensory changes. A person with reconstruction said, “Breast sensation would be wonderful and make a significant difference to aspects of my life. … I can't feel most of my front and it impacts in so many ways—from getting wet when washing up, not noticing shirts being open, to sex.”

Health care professionals were more likely than others to comment specifically on the importance of loss of breast sensation for sexual function. A surgeon responded, “For many breast sensation is extremely important for themselves and their partners!” A nurse said, “Breast sensation and nipple sensation have a role in optimal sexual health … which have affect [*sic*] your sexual function, overall survivorship, and quality of life!”

Several replies highlighted lack of communication between physicians and patients about the loss of sensation after breast cancer surgery. One woman with flap reconstruction said: “I do think it's important to warn. … Warning would have helped come to terms with it … it was a complete shock when I had numbness following sentinel node biopsy pre big op. It was like a slap in the face that this was really happening. I cried in the shower when I realized. Surgeon was like, yes of course there is numbness, but no one warned me.”

A nurse and breast cancer advocate wrote, “If the amount of discussion about it [breast sensation] before surgery is anything to go by, your skeptic is not alone.” A surgeon acknowledged, “Understanding and addressing this in the OR is key!”

Some replies indicated that loss of sensation gave pain relief (“my cancer caused pain and discomfort so I was happy to have a bit less sensation”) and even psychological relief (“They [my breasts] tried to kill me, get rid of them. I want them gone”). Others shared they had regained some or most sensation. One person who had come to accept loss of breast sensation after treatment commented, “It's really important and I wasn't warned well enough. I accept it as part of the wider loss to save my life.”

## Discussion

“It's not clear how important sensation is to patients after mastectomy.”

This study draws on extant data from three sources to further establish that sensation is important to patients after mastectomy. It elucidates, from the patient, advocate, and clinician perspectives, the many mechanisms through which loss of breast sensation affects health and quality of life of women following surgical treatment for breast cancer. It adds that women with and without breast cancer identify several functions of the breast, including breastfeeding, sexual function, and femininity, all of which have been unsatisfactorily addressed by most attempts at breast restoration after cancer treatment. In contrast, surgical innovation to restore the penis lost to cancer or trauma has operated from the premise that appearance, embodiment, and full function of the penis (sexual and urinary) are of very high importance to men and their partners, and are requisite for establishing procedural success.^30,38^ Of note, the literature on male penis reconstruction and transplant is devoid of any discussion of whether or how important penile sensation is to patients contemplating treatment after losing part or all of their penis.^30,39–41^ The universal assumption in this literature is that penile sensory and sexual function are important to men and their sexual partners.

We confirm prior studies in Dutch cohorts that find breast sensation is important to women with and without breast cancer.^8,42^ We also corroborate that, for women with breast cancer, breast sensation is important to quality of life, sexual function, and satisfaction with operative outcome.^8,10–12,43–45^ With a more diverse sample than prior studies (which either do not report race/ethnicity or include mostly white participants) and by adding qualitative data to evidence from close-coded surveys, we add new insight about how breast sensation is important. Loss of breast sensation can interfere with several key domains of sexual function, as well as the relationship with a partner. It was also found to diminish other domains of function, including social and daily activities, and contributed to bothersome symptoms, such as itching, lack of proprioception, and unwanted breast exposure. Although not elicited here, prior studies have documented breast burns and contusions as a result of numbness following mastectomy.^9^

Perhaps the most important, but wholly unsurprising, finding is that loss of breast sensation caused women to feel that an essential body part no longer belonged to them. The importance of successful embodiment has been associated in the literature on amputation and prosthesis—including penile amputation and reconstruction—with improved physical and social functioning, reduced pain, and patient satisfaction.^46–50^ Feelings of disembodiment can be deeply distressing, especially for sexual organs like the breasts or the penis. In fact, one Chinese man had his penis transplant removed due to his inability to accept the organ as his own and his partner's distress.^51,52^

Loss of breast sensation is accepted by some women as the price to pay to be rid of cancer or for survival. Others describe feelings of grief, sorrow, distress, loss, and anger. Lack of patient–physician communication about the effects of mastectomy with reconstruction on breast sensation and the subsequent impact exacerbates negative experiences and is obviously preventable. The skeptic's question resonates with this communication gap—if physicians fail to appreciate the essential functions of the breast, they may very well overlook the importance of these functions to a woman who loses one or both of her breasts to cancer.

While filling gaps in prior work, this study has limitations, including generalizability. First, just as the penile reconstruction literature assumes (rather than empirically establishes) the importance of penile sensation to men, so did we assume the importance of breast sensation to women in designing our studies. Data were obtained from extant data sources, rather than prospectively, and may be limited by selection bias. Last, proportions derived from qualitative data should not be interpreted as prevalence rates of the importance of breast sensation in broader populations. The functions of the breast elicited here, in addition to lactation, are a subset of all functions that might be identified by a larger, prospectively designed survey focused specifically on this question.

## Conclusions

The skeptic's question teaches some lessons. First, evidence for gender bias in medicine and science is ample.^53–57^ We must approach the care and study of women with an understanding of the impacts of these biases on the health, wellbeing, and bodily integrity of women and others. Second, according to the ethical principles of autonomy and nonmalfeasance, interventions to remove, alter, or reconstruct body parts require a conversation with a patient about the full implications.^58^ Informed consent must include educating patients contemplating mastectomy about what to expect from treatment options, including esthetic, functional, and symptomatic sequelae. Last, before depriving a woman of ethical care or a scientist of funding, skeptics of whether a body part is important to women can easily check their bias. Simply replace the word “women” with “men” and “breast” with “penis,” as in: “It's not clear how important penis sensation is to men after penectomy.” The ridiculousness of this statement is eminently obvious and an alert to skeptics to reconsider the fairness of their clinical and scientific judgment.

## Abbreviations Used

IRB: Institutional Review Board
PRISM: University of Chicago Program in Integrative Sexual Medicine for Women and Girls with Cancer
